# Understanding green discoloration in serum and citrate plasma samples: a case report

**DOI:** 10.11613/BM.2025.011001

**Published:** 2024-12-15

**Authors:** Iva Friščić, Sonja Perkov, Mirjana Mariana Kardum Paro

**Affiliations:** Department of Medical Biochemistry and Laboratory Medicine, Merkur University Hospital, Zagreb, Croatia

**Keywords:** case report, sample discoloration, interferences, intensive care, sterile dye

## Abstract

This case report investigates the occurrence of green discoloration in serum and citrate plasma samples collected from a male adult patient following a multivisceral organ transplant. In collected samples, it was necessary to investigate the influence of sample discoloration on the results of laboratory tests and to determine the appropriate approach to sample management. Hematology, coagulation and blood gas analysis showed no flags, but the biochemical lipemia index was susceptible to positive interference, necessitating dilution of the native sample. Despite the green discoloration, both native and diluted samples exhibited minimal interference on routine clinical chemistry analyses, demonstrating the reliability of the laboratory test results. This case report underscores the influence of preanalytical factors on the results of laboratory tests, the need for a thorough assessment of the sample adequacy for laboratory testing and the strict application of appropriate guidelines in the sample management in order to make an accurate diagnosis and ensure optimal patient care.

## Graphical abstract

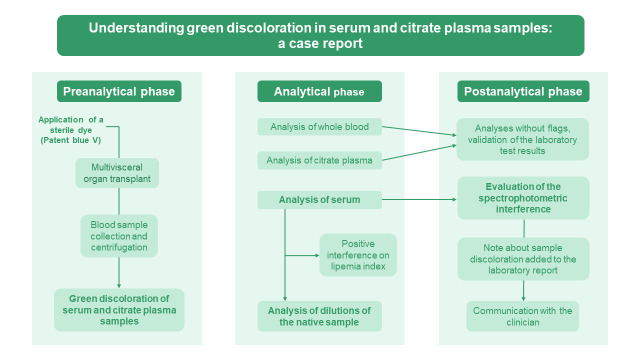

## Highlights

• Green discoloration of serum and plasma samples after sterile dye application

• Positive interference on the lipemia serum index due to the dye’s absorbance

• Evaluation of spectrophotometric interference in native sample dilutions

• No significant spectrophotometric interference on key clinical chemistry tests

• Sample color and potential interference noted in the report and shared with clinicians

## Introduction

The typical color of serum and plasma is a pale yellow, caused by yellow pigments such as bilirubin, carotenoids, hemoglobin and iron transferrin (*1*). Within laboratory settings, it is not uncommon to observe diverse serum and plasma color variations following the centrifugation of whole blood, particularly in specimens obtained from intensive care units and emergency departments. Among these, a significant proportion of samples predominantly display a red color, indicating hemolysis, the rupture of erythrocytes and other blood cells, resulting in the release of cellular components into the serum or plasma (*2*, *3*). The predominant portion of hemolysis occurs *in vitro* and originates from preanalytical errors during phlebotomy, sample transportation, preparation and storage, while hemolysis *in vivo* only accounts for 2-3% of cases (*3*). Furthermore, common causes of sample discoloration include icterus, characterized by a dark yellow to orange serum or plasma color due to high bilirubin concentrations (exceeding 100 µmol/L) and lipemia, presenting as a milky appearance due to higher lipoprotein concentrations (*2*).

Atypical sample coloration has already been described in the literature, starting from dark brown, attributed to intravascular hemolysis, strawberry pink, associated with familial combined hyperlipidemia to green, during the application of contrast dyes, uptake of certain medications such as sulfonamides and higher estrogen concentrations due to oral contraceptive therapy, hormone therapy or pregnancy (*1*, *4*-*10*). This case report outlines the presentation of a patient exhibiting green discoloration observed in citrate plasma and serum samples, as well as the strategies implemented for managing these samples.

## Laboratory analyses

A male adult patient was admitted to the surgical intensive care unit following a multivisceral organ transplant. Following the post-transplant monitoring protocol, blood samples were collected upon admission to the intensive care unit. All blood samples were collected in vacuum tubes supplied by a single distributor (Becton Dickinson and Company, Franklin Lakes, USA). For hematology analysis (complete blood count) whole blood was collected in a K_3_EDTA vacuum tube and for coagulation analysis (prothrombin time (PT), activated partial thromboplastin time (APTT) and fibrinogen) in a sodium citrate (3.2% (0.109 M)) vacuum tube. For routine clinical chemistry analysis (urea, creatinine, bilirubin, total (TBIL) and direct (DBIL), aspartate aminotransferase (AST), alanine aminotransferase (ALT), gamma-glutamyltransferase (GGT), alkaline phosphatase (ALP), C-reactive protein (CRP)) blood was collected in a red cap vacuum tube with micronized silica particles cloth activator. Arterial blood for blood gas, electrolyte (sodium, potassium, chloride and ionized calcium) and lactate analysis was collected in a syringe with spray-dried balanced lithium heparin. Complete blood count was determined on the Sysmex XN1000 hematological analyzer (Sysmex, Kobe, Japan), while arterial blood was analyzed on the blood gas analyzer Radiometer ABL90 FLEX (Radiometer, Bronshoj, Denmark). Both analyses were performed without any problems or flags on the analyzers. After validation of the laboratory test results, laboratory reports were sent to the attending clinicians.

**Figure 1:**
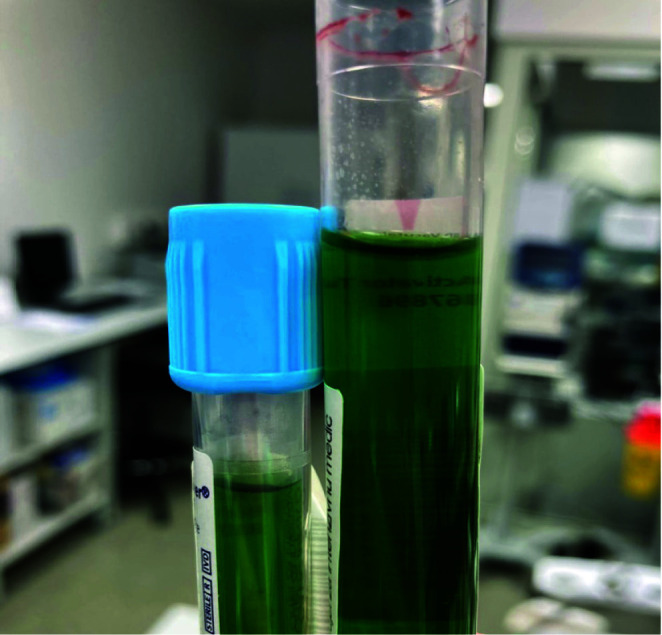
Patient’s blood samples: green discoloration of citrate plasma (left) and serum (right) after centrifugation.

Vacuum tubes for coagulation and clinical chemistry analyses were centrifugated prior to analysis (2100xg for 15 minutes and 2100xg for 10 minutes, respectively). After centrifugation, green discoloration of serum and citrate plasma samples was observed visually (Figure 1). Coagulation analysis was performed on the coagulation analyzer Sysmex CS2500 (Sysmex, Kobe, Japan), equipped with a light source that enables simultaneous multi-wavelength scanning of reactions at 340, 405, 575, 660 and 800 nm, effectively eliminating spectrophotometric interference from the discolored sample. Clinical chemistry analytes were determined on the biochemical analyzer Beckman Coulter DxC700AU (Beckman Coulter, Brea, USA). According to our laboratory protocol, in addition to the tests requested by the attending clinician, hemolysis, icterus and lipemia (HIL) indices are measured for every serum sample as a reflex test using the Beckman Coulter photometric method. These indices are assessed semi-quantitatively, providing an approximate concentration of the interfering substances that cause absorbance changes in the tested samples. Positive interference on the lipemia index was observed, which was attributed to the green discoloration of the sample, as noted visually. To evaluate spectrophotometric interferences, serum dilutions with physiological saline solution (0.9%) were prepared until the semi-quantitative index reached a reference value of zero, indicating the presence of a minimal amount of the interfering substance.

## Further investigation

Biochemical analysis was conducted on the native, undiluted serum sample and two following dilutions were prepared using physiological saline solution (0.9%) (at 75% and 50% of the undiluted sample volume). Alongside the laboratory tests requested by the attending clinician (urea, creatinine, TBIL, DBIL, AST, ALT, GGT, ALT, CRP), additional analysis was performed for clinical chemistry tests commonly requested from the surgical intensive care unit (calcium, inorganic phosphate, magnesium, glucose, uric acid, lactate dehydrogenase (LD), creatine kinase (CK), amylase, lipase, cholesterol, total protein, albumin, myoglobin).

Expected values were mathematically derived from the undiluted serum sample values and bias was calculated for each analyte in every dilution. To evaluate interference, the calculated biases were compared against the maximal allowable bias data from the European Federation of Clinical Chemistry and Laboratory Medicine (EFLM) Biological Variation (BV) Database (*11*). Minimum BV criteria were employed to assess the bias for electrolytes, total protein and albumin, while desirable criteria were applied for all other analytes. No criteria were available for myoglobin. Relevant results are presented in Table 1.

**Table 1:** Laboratory test results in undiluted and diluted serum samples (75% and 50% sample dilution)

**Analyte, method** **(unit)**	**Undiluted serum sample**	**75% sample dilution**			**50% sample dilution**			**Bias: BV criteria** **(%)**
**Expected**	**Measured**	**Bias (%)**	**Expected**	**Measured**	**Bias (%)**
Color	Green	Light green			Light yellow			/
HIL index, photometry (semi-quantitative)	0 0 2	0 0 1			0 0 0			/
Calcium, photometry, arsenazo (III) (mmol/L)	2.51	1.88	1.87	- 0.7	1.26	1.24	- 1.2	1.2
Inorganic phosphate, photometry, molibdate (mmol/L)	0.26	0.20	0.19	- 2.6	0.13	0.14	7.7*	5.8
Magnesium, photometry, xylidyl blue (mmol/L)	0.56	0.42	0.42	0.0	0.28	0.28	0.0	2.4
Glucose, photometry, hexokinase (mmol/L)	9.7	7.3	7.2	- 1.0	4.9	4.8	- 1.0	2.3
Urea, photometry (mmol/L)	5.8	4.4	4.2	- 3.4	2.9	2.7	- 6.9	13.3
Creatinine, photometry, enzymatic (µmol/L)	92	69	68	- 1.4	46	46	0.0	4.4
Uric acid, photometry (µmol/L)	221	166	164	- 1.1	111	110	- 0.5	8.1
Bilirubin, total, photometry, DPD with caffeine (µmol/L)	44	33	33	0.0	22	23	4.5	8.0
Bilirubin, direct, photometry, DPD (µmol/L)	21	16	17	7.9	11	13	23.8*	
Aspartate aminotransferase, photometry (U/L)	862	647	645	- 0.2	431	431	0.0	5.3
Alanine aminotransferase, photometry (U/L)	517	388	382	- 1.5	259	255	- 1.4	9.3
Gamma-glutamyltransferase, photometry (U/L)	11	8	8	- 3.0	5.5	5	- 9.1	11.5
Alkaline phosphatase, photometry (U/L)	20	15	14	- 6.7*	10	10	0.0	5.5
Lactate dehydrogenase, photometry (U/L)	1360	1020	1029	0.9	680	693	1.9	3.1
Creatine kinase, photometry (U/L)	320	240	236	- 1.7	160	157	- 1.9	8.9
Amylase, photometry (U/L)	181	136	135	- 0.6	91	89	- 1.7	6.3
Lipase, photometry (U/L)	114	86	84	- 1.8	57	56	- 1.8	6.6
Cholesterol, photometry (mmol/L)	0.8	0.6	0.6	0.0	0.4	0.4	0.0	4.0
Total protein, photometry, biuret reaction (g/L)	45.7	34.3	33.9	- 1.1	22.9	22.2	- 2.8*	1.6
Albumin, photometry, bromcresol green (g/L)	37.8	28.4	28.1	- 0.9	18.9	18.5	- 2.1*	1.8
C-reactive protein, immunoturbidimetry (mg/L)	20.3	15.2	15.2	- 0.2	10.2	10.6	4.4	23.2
Myoglobin, immunoturbidimetry (µg/L)	551	413	410	- 0.8	276	281	2.0	/
*Biases above the BV criteria. Bias was calculated from the deviation of the measured from the expected value. Obtained biases were compared to biological variation criteria. BV - biological variation. HIL - hemolysis, icterus and lipemia index. DPD - 3,5-dichlorophenyldiazonium tetrafluoroborate.								

All calculated biases were satisfactory, except for the 75% sample dilution for ALP and the 50% sample dilution for inorganic phosphate, DBIL, total protein and albumin. These discrepancies can be attributed to the low expected values of these analytes, which are not clinically significant considering their absolute values (1 U/L for ALP, 0.01 mmol/L for inorganic phosphate, 2 µmol/L for DBIL, 0.7 g/L for total protein and 0.4 g/L for albumin). The minimal differences observed between diluted and undiluted serum samples indicate no significant spectrophotometric interference from the green discoloration in any of these biochemical analyses. Hemolysis, icterus and lipemia indices were determined to be 0 for the H and I index, and 2 for the L index in the undiluted serum sample. The L index decreased by 1 point for each dilution of the serum sample.

## What happened?

Multivisceral organ transplantation is a complex surgical procedure that involves the simultaneous transplantation of multiple abdominal organs, such as the stomach, liver, pancreas, intestine and kidneys. It requires careful visualization of blood vessels and precise identification of vascular structures to ensure proper anastomosis and reduce the risk of complications (*12*). In this case, for the purpose of blood vessel visualization, the patient received an administration of the Patent Blue V sterile dye solution (*13*). The addition of the blue dye solution to the inherent yellow tone of the serum and plasma results in a cumulative green discoloration, a phenomenon that persists for 24-48 hours, corresponding to the elimination half-life of the sterile dye (*8*, *13*).

The change of sample color and thus the presence of the interfering substance was noticed visually and by the higher L index. The observed higher L index was not attributable to serum lipemia but to the specific wavelengths at which the Beckman Coulter HIL reagent measures serum indices. Specifically, wavelengths used for the H index are 410 and 480 nm, for the I index 480 and 570 nm, and for the L index 660 and 800 nm. The absorbance peak of Patent Blue V occurs at 640 nm, which overlaps with the wavelength used for L index measurement, thereby causing a positive interference (*14*). However, diluting the serum samples with physiological saline solution effectively reduced this positive interference, thereby minimizing its impact on the L index. Among the laboratory tests investigated, the absorbance peaks closest to the dye’s absorbance peak are found in the measurements for calcium (photometry, arsenazo III), which is measured bichromatically at 660 and 700 nm and uric acid (photometry), measured bichromatically at 660 and 800 nm. For these analytes, no significant bias was detected relative to the biological variation criteria between the diluted samples and the native specimens, indicating that sample discoloration had minimal spectrophotometric impact on laboratory test results. Because of the possibility of other sources of interference caused by the dye, a comment about sample discoloration and potential interference on the laboratory test results was added to the laboratory report.

## Discussion

Green serum and citrate plasma samples are a relatively uncommon phenomenon encountered in laboratory practice, originating from various factors such as estrogen therapy, medication interference or contamination. The incidence of encountering these samples depends on various factors, including patient demographics, underlying health conditions, sample-handling protocols and the specific assays performed (*8*). The green discoloration may not always be immediately noticeable or easily explained without further investigation. Although rare in most laboratory settings, the presence of green serum and citrate plasma samples requires comprehensive assessment and appropriate measures to ensure the accuracy of laboratory test results. Increased awareness of this preanalytical variability is essential to reduce the risk of improper sample handling, incorrect sample rejection and potential delays in sample processing and patient care (*1*).

Sample dilution is a method that can help minimize spectrophotometric interference from serum discoloration but cannot eliminate other sources of interference caused by the interferent itself, such as chemical interference with the analyte or reagent. To investigate other potential sources of interference, it is recommended to obtain another sample from the same patient without the interferent or to spike another sample with the interferent and compare the results before and after spiking.

Furthermore, communication with the attending clinician is essential, as the clinician can provide important information regarding the use of known discoloring substances, while the laboratory specialist can offer insights on possible interferences in test results. A comment about sample discoloration and potential interference should be a mandatory component of the written laboratory report.

## What YOU should / can do in your laboratory to prevent such errors

The green discoloration observed in serum and citrate plasma samples resulting from the sterile dye Patent Blue V has minimal spectrophotometric interference on the outcomes of clinical chemistry test results. However, since spectrophotometric interference is not the only potential source of interference and this dye may not be the sole factor causing the green discoloration in serum and citrate plasma samples, further investigation is required. If possible, the first step in the follow-up investigation should be to obtain a sample without interference. If that is not an option, it is advisable to perform biochemical tests on both undiluted and diluted serum samples. Adding a comment about the sample color in the laboratory report and discussing these observations and possible interferences with the clinician are recommended steps to ensure a thorough understanding and accurate interpretation of laboratory test results.

## Data Availability

All data generated and analyzed in the presented study are included in the published article.
